# Oncocytoma managed by active surveillance in a transplant allograft kidney: a case report

**DOI:** 10.1186/s12957-018-1426-2

**Published:** 2018-07-02

**Authors:** Duilio Pagano, Fabrizio di Francesco, Liotta Rosa, Chibueze A. Nwaiwu, Sergio Li Petri, Salvatore Gruttadauria

**Affiliations:** 1 Department for the Treatment and Study of Abdominal Diseases and Abdominal Transplantation, IRCCS-ISMETT (Istituto di Ricovero e Cura a Carattere Scientifico - Istituto Mediterraneo per i Trapianti e Terapie ad alta specializzazione), UPMC (University of Pittsburgh Medical Center) Italy, Via E. Tricomi 5, 90127 Palermo, Italy; 2 Department of Diagnostic and Therapeutic Services, IRCCS-ISMETT, UPMC Italy, Palermo, Italy; 3 University of Pittsburgh School of Medicine, Pittsburgh, PA USA

**Keywords:** Kidney transplantation, Renal transplantation, Solid renal mass, Oncocytoma

## Abstract

**Background:**

The ethical implications of the utilization of kidneys with solid renal masses (SRMs) in transplantation are the subject of lively debate in the transplantation community and beyond. One of such implications is that as the life expectancy of renal transplant patients improve, the prevalence of SRMs in donors is likely to increase. We report a case of an oncocytoma in a renal allograft complicating a deceased-donor kidney transplant.

**Case presentation:**

A 60-year-old woman received and underwent deceased-donor renal transplantation for end-stage renal disease after a waiting-list period of 11 years. Kidney Doppler ultrasound (DUS) of the deceased donor was negative for any nodular lesion. The finding of the DUS, done on postoperative day 1, to assess the patency of the graft, was suspicious for an acute arterial thrombosis but did not reveal any focal irregularities. An ensuing computed tomography (CT) scan did not show any arterial complications but serendipitously revealed a 2.4-cm lesion on the upper pole of the renal allograft, which was not detected during the back-table or ultrasonography monitoring. Histology of the biopsied lesion was consistent with oncocytoma. However, because the eosinophilic variant of chromophobe renal cell carcinoma may morphologically resemble renal oncocytoma, immunohistochemical staining was performed. The results were negative, ruling out chromophobe RCC. After discussing the therapeutic options and potential related outcomes with the patient, we found no reason for resection of the lesion or an allograft nephrectomy, given the low risk of malignant transformation in an oncocytoma. Active surveillance of the benign tumor was done with ultrasonography, every 2 months, for the first year and, then, with magnetic resonance imaging, every year. The patient received mycophenolate-mofetil, tacrolimus, and prednisone throughout the 5-year follow-up period, and the regimen for immunosuppression was not changed despite the presence of the renal mass. After 60 months, we report that none of the radiological findings have shown any morphological changes of the lesion, and the patient is well.

**Conclusion:**

To the best of our knowledge, we report the first case of an oncocytoma in a renal allograft complicating a deceased-donor kidney transplant, which was successfully managed by active surveillance.

## Background

The ethical implications of the utilization of kidneys that have been found to have solid renal masses (SRMs) in transplantation are the subject of lively debate in the transplantation community and beyond. One of such implications is that as the life expectancy of renal transplant patients improve, the prevalence of SRMs in donors is likely to increase [1, 2]. A recent review of the literature on SRMs in transplant allograft kidneys proposed a detailed management algorithm for the tumors in allograft kidneys that mirrors the clinical decision-making in the non-transplant population [3]. We report a case of an oncocytoma in a renal allograft complicating a deceased-donor kidney transplant in a 60-year-old woman.

## Case presentation

**Fig. 1:**
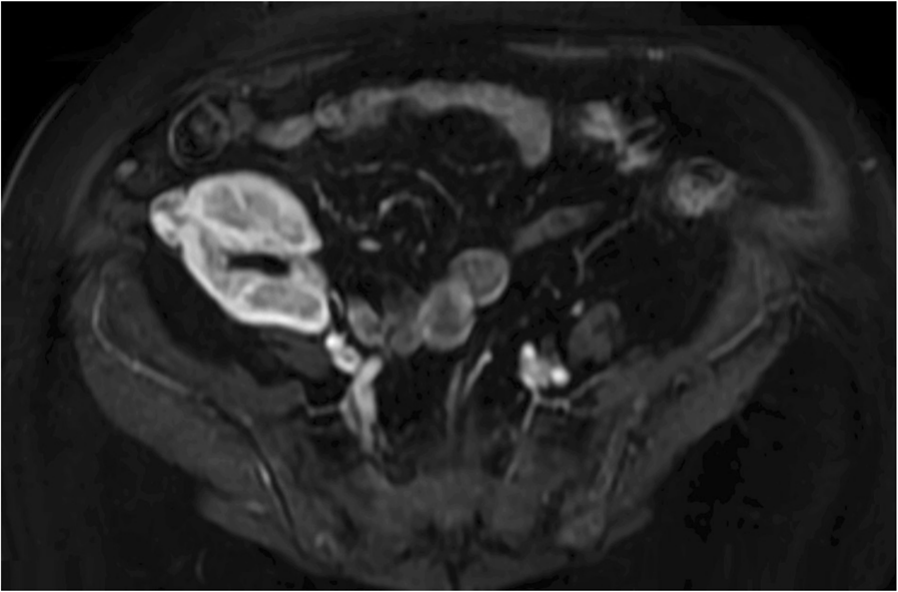
Magnetic resonance imaging (MRI) was used for the follow-up evaluation of renal mass because of its multi-planar capabilities, as well as its ability to demonstrate of enhancement and to provide soft-tissue contrast. A MRI scan at 60 months after transplantation showed a well-circumscribed oncocytoma with expansive net margins at the cortical surrounding of the kidney (white arrows). It has not infiltrative margins with extension in peri-renal fat or renal sinus, in the medulla of the kidney or in the major renal venous vessels

**Fig. 2:**
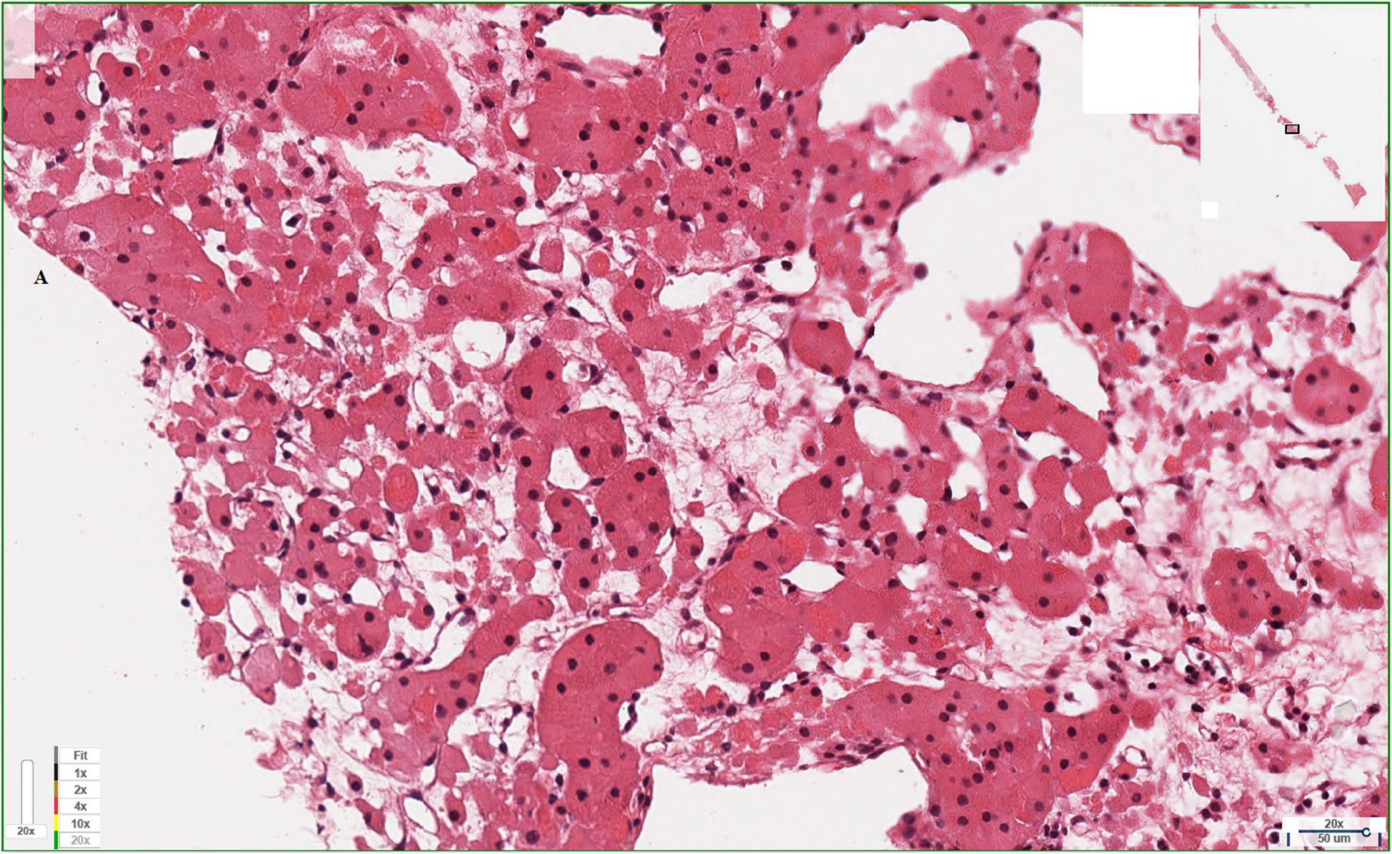
Histological examination showed small islands and microcysts of large, mostly round, eosinophilic cells (oncocytes) within hypocellular loose connective tissue, consistent with oncocytoma. Renal cell carcinoma has been ruled out

The deceased donor was a 67-year-old man with a kidney Doppler ultrasound (DUS) that was negative for any nodular lesion. As part of the routine postoperative follow-up management, the recipient underwent DUS to assess the patency of the graft on postoperative day 1. The DUS finding was suspicious for an acute arterial thrombosis but did not reveal any focal irregularities. Consequently, a computed tomography (CT) scan was urgently obtained but it did not show any arterial complications. However, it serendipitously revealed a 2.4-cm lesion on the upper pole of the renal allograft which was not detected during the back-table or ultrasonography monitoring. A biopsy of the lesion was performed, and its histology revealed an epithelial proliferation of large cells with finely granular cytoplasm and medium round nucleus vesicular acidophilus, arranged tubules, and alveoli and cords immersed in a connective tissue stroma. This picture was consistent with oncocytoma. However, because the eosinophilic variant of chromophobe renal cell carcinoma (RCC) may morphologically resemble renal oncocytoma, immunohistochemical staining was performed using Ki-67 antibodies and RCC antigens. The results were negative, ruling out chromophobe RCC. The therapeutic options and potential related outcomes were clearly discussed with the patient. Given the low risk of malignant transformation in an oncocytoma [4], we found no reason for resection of the lesion or an allograft nephrectomy. Consequently, we opted for active surveillance of the benign tumor with ultrasonography, every 2 months, for the first year and, then, with magnetic resonance imaging (MRI), every year (Fig. 1). The patient received mycophenolate-mofetil, tacrolimus, and prednisone throughout the 5-year follow-up period and the regimen for immunosuppression was not changed despite the presence of the renal mass. After 60 months of active surveillance, we report that radiological studies have shown no growth, regression, or any other interim morphological changes to the lesion, and the patient is alive and well (Fig. 2).

## Discussion

Renal oncocytomas are considered benign kidney tumors [5] with an incidence of 3–7% among all solid renal neoplasms [6]. They are often diagnosed incidentally as SRMs and have traditionally been managed by nephrectomy as it is often suspected to be chromophobe RCC [6]. Treatment results are excellent, with only one case of metastatic tissue proven as oncocytoma reported in the literature [7, 8]. Thus, making a correct preoperative diagnosis of renal oncocytoma is vital as it can allow for the prevention of unnecessary and aggressive surgical procedures in both transplant and non-transplant patients. A recent study in the UK on the surgical management of renal oncocytomas showed a 0.4% 60-day mortality rate and a 20% in-hospital complication rate, including bleeding, pneumothorax, chest infection, splenectomy, wound infection, ileus, and deep vein thrombosis/pulmonary embolus [9]. Furthermore, an accurate preoperative diagnosis could be of benefit when an SRM is detected during living kidney donor evaluation or is found in kidneys from deceased donors. However, cohort studies are needed to better understand the effect of the active surveillance approach in the management of asymptomatic renal oncocytoma in the transplant population. This could, potentially, help alleviate some of the burden of an increasing demand for kidneys for transplantation as kidneys that were once abandoned could be put into good use in the right patients.

There are case reports of coexistent RCC associated with or even within oncocytomas, and we are not aware of any reports indicating that immunosuppressive regimen facilitates oncocytoma malignant transformation [4, 10]. Nevertheless, we recommend that clinicians remain mindful that the growth patterns of renal oncocytoma and those of RCC can be similar. Transparency and clinical clarity are two cornerstones in this act of sharing experiences with the medical community at large. To the best of our knowledge, this is the first case report of oncocytoma managed by active surveillance in a transplant allograft kidney. Whether this scarcity in the literature reflects the rarity of the clinical phenomenon or a reluctance to report it when detected is not for our institute to speculate [3]. We would suggest to consider this clinical entity as a non-surgical oncologic disease, believing that although the risk of malignant transformation of oncocytoma is low in the non-transplant population, awareness of the risk and the use of appropriate protocols for informed consent and surgical decision-making is paramount for continued success in this delicate field of medicine.

## Conclusion

We report a case of an oncocytoma in a renal allograft complicating a deceased-donor kidney transplant in a 60-year-old woman, managed by active surveillance.

## Abbreviations

CT: Computed tomography
DUS: Doppler ultrasound
MRI: Magnetic resonance imaging
RCC: Renal cell carcinoma
SRM: Solid renal mass
